# How COVID-19 Information Fear of Missing out Increases the Risk of Depression and Anxiety: Roles of Resilience and Personality Types

**DOI:** 10.3390/bs14050359

**Published:** 2024-04-25

**Authors:** Yuling Tang, Binbin Wang, Chunyan Xu, Xiaochun Xie

**Affiliations:** 1 School of Psychology, Northeast Normal University, Changchun 130024, China; tangyl296@nenu.edu.cn (Y.T.); xucy032@nenu.edu.cn (C.X.); 2 CAS Key Laboratory of Mental Health, Institute of Psychology, Chinese Academy of Sciences, Beijing 100101, China; wangbb@psych.ac.cn; 3 Department of Psychology, University of Chinese Academy of Sciences, Beijing 100049, China; 4 Research Center of Mental Health Education in Northeast Normal University, Key Research Institute of Humanities and Social Science in Universities in Jilin Province, Changchun 130024, China

**Keywords:** COVID-19, fear of missing out, depression, anxiety, resilience, personality types

## Abstract

During major health emergencies (e.g., the COVID-19 pandemic) people often fear missing relevant information. COVID-19 information fear of missing out (FOMO) is a phenomenon where people feel anxiety about losing control of COVID-19-related information. The present study aimed to examine how COVID-19 information FOMO relates to mental health (e.g., depression and anxiety), the mediating role of resilience, and the moderating role of personality types during the COVID-19 pandemic. We surveyed 1442 Chinese undergraduates (*M*_age_ = 21.68 ± 2.35 years) on the relevant variables. The results showed that COVID-19 information FOMO was positively associated with depression and anxiety, and resilience mediated these associations. Latent profile analysis (LPA) identified three personality types (undercontrolled, adaptive, and overcontrolled). Personality types moderated the mediation models, in which the indirect effects were only significant in the participants classified in the undercontrolled group rather than the participants classified in the other two groups. This study told us that undergraduates’ mental health, particularly that of the undercontrollers, should be paid attention to when responding to a major public health emergency (e.g., the COVID-19 pandemic).

## 1. Introduction

Coronavirus disease 2019 (COVID-19) caused a global public health crisis and harmed public mental health. During this pandemic, people have paid more attention to COVID-19-related information, leading to an increase in reporting on the practical constraints of life in lockdown and an increase in the demand for up-to-date knowledge due to feelings of uncertainty [1]. COVID-19 information fear of missing out (FOMO) is a new concept, developed by Yu et al. (2020), to describe this phenomenon. COVID-19 information FOMO is defined as a negative condition in which people feel anxious about losing control over COVID-19-related information [2], and it is based on traditional social FOMO. Social FOMO is “a pervasive apprehension that others might be having rewarding experiences from which one is absent” [3], (p. 1814). These two FOMOs are similar but have differences in their focus. The similarity is that the two FOMOs are both derived from a fear of missing out on important information and opportunities, correlated with stronger social media engagement [4,5]. However, social FOMO focuses on social information and opportunities, while COVID-19 information FOMO focuses on pandemic-related information, preventive health opportunities, and other pandemic-affected plans [2].

As far as we know, previous studies about COVID-19 information FOMO were not adequate since the concept was proposed relatively recently [2,5]. Recently, Koban et al., in 2022, found in a longitudinal study that COVID-19 information FOMO positively predicted daytime tiredness [5]. That study revealed that people’s limited energy resources during lockdown may be severely depleted by the persistently elevated anxiety about losing control of certain information. It implied that COVID-19 information FOMO had potential adverse effects on mental health. However, the above study directly tested neither the relationship between COVID-19 information FOMO and mental health nor the potential mediators (e.g., resilience) and moderators (e.g., personality types) underlying this relationship.

Resilience is a protective factor that mediates and relieves the effect of negative conditions (e.g., stress) on mental health [6]. Personality is associated with a person’s mental health and well-being [7]. Personality types integrally and systematically reflect individual differences in thinking, actions, attitude, and belief [8,9,10]. Therefore, the present study aimed to test the mediating role of resilience and to analyze the moderating role of personality types on the association between COVID-19 information FOMO and depression and anxiety during the pandemic.

### 1.1. The Relationships between COVID-19 Information FOMO and Depression and Anxiety

Depression and anxiety are both negative emotional symptoms. Unsurprisingly, depression and anxiety symptoms increased after COVID-19 broke out [11,12,13]. During the pandemic, people reported higher depression and anxiety scores than the time before the pandemic broke out [14]. Thus, it is necessary to pay attention to issues related to depression and anxiety in the context of the COVID-19 pandemic.

Previous evidence has pointed out that COVID-19 information FOMO is one of the potential risk factors for depression and anxiety. On the one hand, receiving too much COVID-19 information increases the levels of depression and anxiety [15,16,17]. On the other hand, social FOMO is positively related to depression and anxiety [18,19]. Given the similar characteristics between social FOMO and COVID-19 information FOMO, we have the confidence to suppose there is a positive relationship between COVID-19 information FOMO and depression and anxiety. Additionally, COVID-19 information FOMO is linked to daytime tiredness, which is associated with depression and anxiety [5]. Thus, based on the evidence above, we put forward the following hypothesis:

**H1.**  *COVID-19 information FOMO positively relates to depression (H1a) and anxiety (H1b).*

### 1.2. The Mediating Role of Resilience

Although COVID-19 information FOMO may directly relate to depression and anxiety, people with different capacities (e.g., resilience) can cope effectively with COVID-19 information and bounce back from COVID-19 information FOMO. Further, the importance of resilience lies in its action as a mediator between negative factors (e.g., perceived stress, bullying) and mental health [6,20]. Given that COVID-19 information FOMO is one of the negative factors, we assumed that resilience mediated the relationship between COVID-19 information FOMO and depression and anxiety.

Resilience is defined as a dynamic process of positive adaptation in the face of significant adversity [21]. Resilience is an important factor in COVID-19 pandemic-related consequences [22]. Furthermore, resilience is negatively associated with depression and anxiety [6,23,24]. Therefore, we supposed that resilience was negatively linked to depression and anxiety.

Although no study has directly tested the link between COVID-19 information FOMO and resilience, some indirect evidence may suggest a potential relationship. Negative environments and psychosocial conditions (e.g., stressful COVID-19-related life events and pandemic fatigue) decrease individuals’ resilience [25,26]. A relative study on social FOMO revealed that undergraduates with a high level of fear of missing out on information about friends report a low level of resilience [27]. Based on this indirect evidence, we supposed a negative relationship between COVID-19 information FOMO and resilience. Additionally, resilience mediates the relationships between being bullied and psychological stress and mental health [20,24], therefore, we put forward the following mediation hypothesis:

**H2.**  *Resilience mediates relations between COVID-19 information FOMO and depression (H2a) and anxiety (H2b).*

### 1.3. The Moderating Role of Personality Types

Although COVID-19 information FOMO may relate to mental health through the mediating role of resilience, the mediation may vary for different people. The personality processes model revealed that personality types may moderate the relationships between COVID-19 information FOMO and depression and anxiety [28]. The previous studies on personality can be divided into two approaches [29]—a variable-centered approach [30,31,32] and person-centered approach [33,34,35]. When comparing the two, Donnellan and Robins (2010) considered that the typological approach shifts attention to the ways that traits are organized and integrated within individuals [36]. It is a whole individual, instead of isolated traits, that engages in dynamic transactions with COVID-19 information. Therefore, we used a person-centered approach to conduct our research.

Based on the theory of ego-resiliency and ego-control, Block and Block (1980) first identified three common personality types: resilient, overcontrolled, and undercontrolled [33,36,37,38]. Specifically, resilients reflect low neuroticism, high conscientiousness, moderate to high agreeableness, high openness, and high extraversion; overcontrollers tend to have high agreeableness, low extraversion, and low neuroticism; and undercontrollers mainly report low agreeableness and low conscientiousness [38]. According to previous studies, we proposed the following hypothesis:

**H3.**  *Undergraduates’ personality can be divided into three types: resilient, undercontrolled, and overcontrolled.*

Social FOMO is positively associated with neuroticism [39,40,41], but negatively correlated with extraversion, openness, agreeableness, and conscientiousness [40]. Given that personality types are classified by different personality traits, people with the resilient personality type may have the lowest levels of social FOMO among people with these three personality types. It is implied that people with a resilient personality type, compared with the other two personality types (undercontrolled and overcontrolled), may have the lowest levels of COVID-19 information FOMO.

In addition, previous research on personality types has shown that resilients seem to be generally well-adjusted in many respects (e.g., high life satisfaction and prosocial behavior, low levels of psychological distress, and few mental health problems), while both overcontrollers and undercontrollers seem to be less well-adjusted [8,42,43]. That is, resilients have the highest levels of resilience and the lowest levels of depression and anxiety among people with the three personality types.

Although no study directly examines the moderation effect of personality types on the relationships among COVID-19 information FOMO, resilience, and depression, some indirect evidence suggests a potential relationship [44,45,46]. A longitudinal study found that personality types (resilient, average, and oversensitive) moderated the relationship between work stress and life satisfaction [46]. Specifically, the oversensitive profile promoted the negative effect of work stress on life satisfaction, while the resilient profile prevented this negative effect and promoted the positive top-down spillover from life satisfaction to work stress [46]. In addition, personality traits can be a moderator to explain the association between risk factors (e.g., daily hassles, evening chronotypes, and social media exposure) and mental health [44,47,48,49]. For example, individuals with high neuroticism tended to have a stronger relationship between qualitative job insecurity and mental health complaints than individuals with low neuroticism [45]. Thus, the current study aimed to test whether individuals’ personality types moderated the influences of COVID-19 information FOMO on depression and anxiety, and the effects of resilience on depression and anxiety. Based on the above findings, we put forward the other hypothesis:

**H4.**  *The personality types can serve as a moderator variable among the links between COVID-19 information FOMO, resilience, depression (H4a), and anxiety (H4b).*

### 1.4. The Current Study

In sum, previous studies did not examine the role of resilience and personality types on the association between COVID-19 information FOMO and mental health. Some direct and indirect evidence implied that resilience and personality types may play a role in the relationships between COVID-19 information FOMO and depression and anxiety. Therefore, this study aimed to examine the mediation of resilience and to analyze the moderating effect of personality types on the relationships between COVID-19 information FOMO and depression and anxiety during the pandemic.

## 2. Materials and Methods

### 2.1. Participants and Procedure

This study was conducted in the context of the regular prevention and control of COVID-19 in China, and an online questionnaire survey was administered to university students in mainland China through the Questionnaire Star platform in April 2022. We adopted the convenient sampling method. All participants took part in the survey voluntarily and each participant was paid RNB CNY 5 after completing the online questionnaire. A total of 1580 questionnaires were returned, and 1442 valid questionnaires (91.3%) were finally obtained through the following screening conditions: three attention screening questions, a response time of less than 180 s, and use of the careless package [50] of *R* software 4.2.1 [51] to calculate the maximum long-string value and remove the data of subjects who did not answer seriously [52,53]). Participants (*N* = 1442; mean age = 21.68 years; *SD* = 2.35) included 695 males (48.2%) and 747 females (51.8%). We adopted G*Power 3.1 to calculate the posterior sample power of this study, using the minimum correlation coefficient (0.38) as the effect quantity, setting α = 0.05, total sample size = 1442, and finally obtaining the power value (1 − β) = 1.

### 2.2. Measures

#### 2.2.1. COVID-19 Information FOMO

We measured COVID-19 information FOMO using a scale with three items [5]. Participants rated the questions (e.g., When I miss the latest news about the novel coronavirus or hear about it later than others, I get annoyed.) on a five-point Likert scale from 1 = “strongly disagree” to 5 = “strongly agree”. In this study, we first translated the original English scale to Chinese, then asked an associate professor of psychology to revise it, and finally formed the Chinese scale. The results of confirmatory factor analysis showed that the fit indices-CFI/TLI = 1 and all factor loadings ranged from 0.75 to 0.89, reaching the significance level (*p*s < 0.001), which indicated that the structural validity of the model was good. Cronbach’s α = 0.86 was used for the scale.

#### 2.2.2. Connor–Davidson Resilience Scale (CD-RISC-10)

Resilience was assessed using the 10-item short form of the CD-RISC-10 [54,55]. Participants rated the items (e.g., Able to adapt to change.) on a four-point scale ranging from 1 = “Completely not true” to 4 = “Completely true”. We revised this scale into a Chinese version in the current study. In this study, Cronbach’s α = 0.84 was used for this scale.

#### 2.2.3. Chinese Big Five Personality Inventory-15 (CBF-PI-15)

CBF-PI-15 was applied to assess participants’ personality [56]. This scale consists of 15 items with five dimensions (e.g., I often worry about trifles.). Participants were asked to indicate the items on a six-point Likert scale ranging from 1 = “Completely not true” to 6 = “Completely true”. In the present study, Cronbach’s alpha coefficients for neuroticism, conscientiousness, agreeableness, openness, and extraversion were 0.82, 0.70, 0.76, 0.82, and 0.54, respectively.

#### 2.2.4. Depression Anxiety Stress Scale-21 (DASS-21)

Depression and Anxiety were measured by the Chinese version of DASS-21 [57]. The origin DASS-21 consists of 21 items with three dimensions—depression, anxiety, and stress. Each dimension consists of seven items. In the current study, we adopted 14 items for measuring depression (e.g., I no longer seem to have any pleasant, comfortable feelings) and anxiety (e.g., I feel dry in the mouth). Participants rated the items on a four-point scale ranging from 1 = “Never” to 4 = “Always”. In this study, Cronbach’s alpha coefficients for depression and anxiety were 0.91 and 0.89, respectively.

### 2.3. Data Analyses

SPSS 22.0 and Mplus 8.3 were used in the analyses. First, descriptive statistics and Pearson correlation analysis were performed to analyze the associations among COVID-19 information FOMO, resilience, personality, depression, and anxiety. Second, the present study used the bootstrap method (5000 times) to investigate the 95% bootstrap confidence interval (CI) of the indirect effects of structural equality modeling. The mediating effect was significant if the interval did not include zero.

Third, latent profile analysis (LPA) was utilized to identify personality types. Before the LPA, we converted the scores of each personality trait into z-scores [9]. We identified the final appropriate model according to the following criteria: Akaike Information Criterion (AIC), Bayesian Information Criterion (BIC), Sample Size-Adjusted Bayesian Information Criterion (SSABIC), Lo–Mendell–Rubin Likelihood Ratio (LMR-LRT), Bootstrap Likelihood Ratio (BLRT), and entropy. The better model was considered to have (1) smaller comparative values of AIC, BIC, and SSABIC, (2) a statistically significant value for LMR-LRT and BLRT, as well as (3) a larger value of entropy [35,58]. Finally, model comparison and multiple-group analyses were conducted to explore the role of personality types on the relationships between these variables.

The current study used the maximum likelihood estimation to calculate the goodness-of-fit indices, which were adopted to assess the adequacy of model fit, including χ^2^, *df*, Comparative Fit Index (CFI), Tucker Lewis Index (TLI), Standardized Root Mean Square Residual (SRMR), and Root Mean Square Error of Approximation (RMSEA). The cut-off standards of these fit indices are as follows: CFI and TLI values > 0.90 are accepted, and RMSEA ≤ 0.08 are accepted [59]. ΔAIC and ΔSSABIC were used to compare the structure models. The difference between models is significant when the values of ΔAIC and ΔSSABIC are greater than 10 [60].

## 3. Results

### 3.1. Descriptive Statistics

Correlations, means, and Standard Deviations (SD), for all the variables are presented in Table 1. The results show that COVID-19 information FOMO was positively associated with depression (*r* = 0.38, *p* < 0.001) and anxiety (*r* = 0.43, *p* < 0.001), and resilience was negatively associated with depression (*r* = −0.21, *p* < 0.001) and anxiety (*r* = −0.14, *p* < 0.001) (see Table 1).

### 3.2. Mediating Effects of Resilience

This study adopted a two-step approach to test the two mediation models using structural equation modeling [61]. The mediation models illustrated how resilience mediated the relationships between COVID-19 information FOMO and depression and anxiety, respectively. Gender and age were included as covariates in data analyses.

#### 3.2.1. Resilience Mediates the Relationship between COVID-19 Information FOMO and Depression

**Figure 1:**
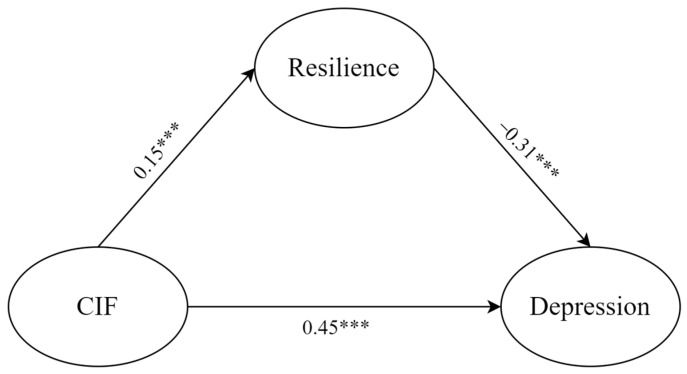
The mediating effect of resilience in the relationship between COVID-19 information FOMO and depression. CIF: COVID-19 information FOMO. *** *p* < 0.001.

The present study first tested how resilience mediated the relationship between COVID-19 information FOMO and depression (Figure 1). In the first step, we examined the measurement model. The measurement model was established with three latent variables (COVID-19 information FOMO, resilience, depression) and twenty observed variables. The fit indices showed a good model fit: χ^2^ = 893.38; *df* = 167; *p* < 0.001; CFI = 0.94; TLI = 0.93; RMSEA = 0.06 with 90%*CI* = [0.05, 0.06]; SRMR = 0.05.

In the second step, structural equation modeling was used to explore the relationship between COVID-19 information FOMO and depression, as well as the mediating role of resilience. The established model was found to fit well (χ^2^ = 1026.82; *df* = 203; *p* < 0.001; CFI = 0.93; TLI = 0.93; RMSEA = 0.05 with 90%*CI* = [0.05, 0.06]; SRMR = 0.05). The results showed that COVID-19 information FOMO positively predicted resilience (β = 0.15; *p* < 0.001; 95%*CI =* [0.09, 0.21]) and depression (β = 0.45; *p* < 0.001; 95%*CI* = [0.39, 0.50]), while resilience had a significant negative association with depression (β = −0.31; *p* < 0.001; 95%*CI* = [−0.37, −0.25]).

The bootstrap method was used to obtain the 95%*CI* of the model path, and the direct and indirect effects of the mediation path are shown in Table 2. It is an unexcepted founding that the direct and indirect effects of COVID-19 information FOMO had an opposite direction; specifically, COVID-19 information FOMO had a positive direct effect on depression, while the indirect effect was negative. These results suggest that resilience is a suppressed mediator. That is, higher levels of COVID-19 information FOMO predicted higher resilience, resulting in lower levels of depression. According to Wen and Ye’s (2014) suggestion, we used the |ab/c’| to indicate the relative mediation ratio in the suppressed mediation model [62]. The results showed that if the |ab/c’| was 11.1%, depression was the outcome.

#### 3.2.2. Resilience Mediates the Relationship between COVID-19 Information FOMO and Anxiety

**Figure 2:**
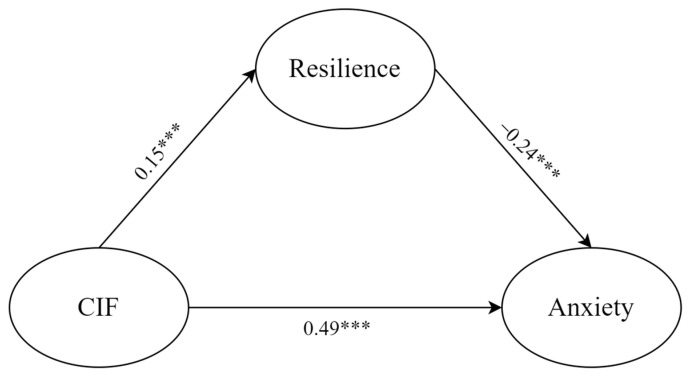
The mediating effect of resilience in the relationship between COVID-19 information FOMO and anxiety. CIF: COVID-19 information FOMO. *** *p* < 0.001.

We then examined how COVID-19 information FOMO related to anxiety through resilience (Figure 2). First, we examined the measurement model, which was established with three latent variables (COVID-19 information FOMO; resilience; anxiety) and twenty observed variables. The fit indices showed a good model fit: χ^2^ = 883.66; *df* = 167; *p* < 0.001; CFI = 0.94; TLI = 0.93; RMSEA = 0.06 with 90%*CI* = [0.05, 0.06]; SRMR = 0.05.

Second, we utilized structural equation modeling to explore the association between COVID-19 information FOMO, resilience, and anxiety. The established model was found to fit well (χ^2^ = 1010.06; *df* = 203; *p* < 0.001; CFI = 0.94; TLI = 0.93; RMSEA = 0.05 with 90%*CI* = [0.05, 0.06]; SRMR = 0.06). As expected, all paths in the model were significant. The results showed that COVID-19 information FOMO positively predicted resilience (β = 0.15; *p* < 0.001; 95%*CI* = [0.09, 0.21]) and anxiety (β = 0.49; *p* < 0.001; 95%*CI* = [0.43, 0.54]), while resilience had a significant negative link with anxiety (β = −0.24; *p* < 0.001; 95%*CI* = [−0.30, −0.18]).

The direct and indirect effects of this mediation path are shown in Table 2. Similar to the mediation model of depression as the outcome, the results showed that the direct and indirect effects of COVID-19 information FOMO had an opposite direction. Specifically, COVID-19 information FOMO had positive direct effects on anxiety, while the indirect effect was negative. These results suggest that resilience was a suppressed mediator. That is, a higher level of COVID-19 information FOMO predicted higher resilience, leading to lower anxiety. The results showed that the |ab/c’| had to be 8.2% for anxiety to be the outcome.

### 3.3. The Personality Profiles

**Figure 3:**
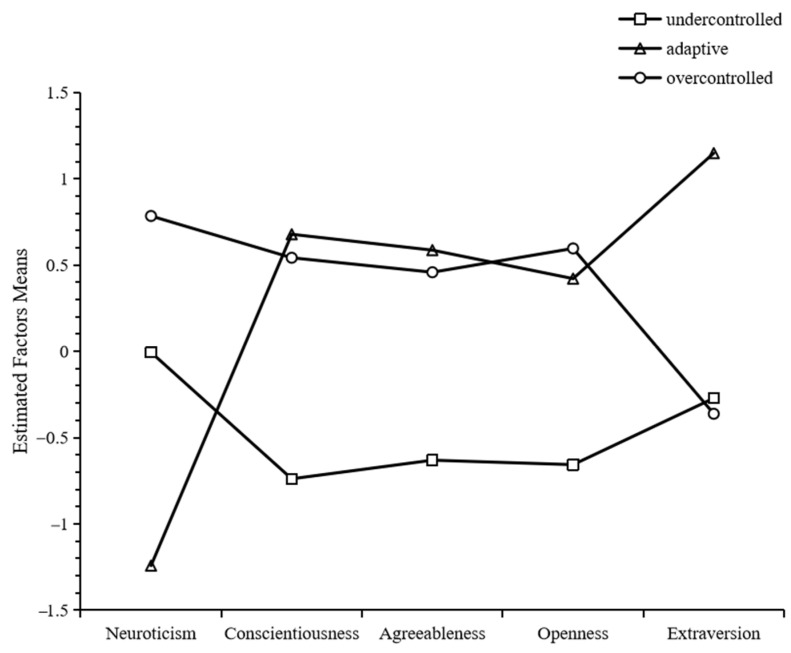
The latent profile of college students’ personality types.

We used LPA models to identify the personality types by classifying five personality traits. As Table 3 showed, the three-class model had lower AIC, BIC, and SSABIC values than the two-class model and had significant *p*-values of LMR-LRT and BLRT (*p*s < 0.001). This indicated that the three-class model was better than the two-class model. The four-class model had lower AIC, BIC, and SSABIC values than the three-class model, and had a significant *p*-value of BLRT (*p* < 0.001), but had no significant *p*-value of *p* for LMR-LRT (*p* > 0.05). That is, the four-class model explained no more of the variance than the three-class model in terms of the personality profile. Additionally, Li et al. (2017) suggested that the participant number in each subgroup should not be less than 5% of the whole sample or fewer than 30 [63]. In the four-class model, one of the classes (*n* = 29, 2.0%) was not content with this requirement. In addition, considering the simplicity of the model, the three-class model was chosen as well (Figure 3).

The current study determined three personality types in Chinese undergraduates during COVID-19 closely resembling those found by Robins et al. (1996) [38]. We adopted standard deviation to identify the personality types according to the following criteria: a score equal to or larger than 0.5 represented a high score; a score between −0.5 and 0.5 was a moderate score; and a score equal to or less than −0.5 represented a low score. Therefore, the first type (*n* = 643, 44.6%) was characterized by moderate neuroticism and extraversion, and low conscientiousness, agreeableness and openness, and labeled as undercontrolled. The second type (*n* = 318, 22.1%) was characterized by high conscientiousness, agreeableness and extraversion, and low neuroticism and openness. To discern resilience and one of the personality types in the present study, this type was labeled adaptive, instead of resilient. The third type (*n* = 481, 33.4%), referred to as overcontrolled, was characterized by high neuroticism, conscientiousness and openness, and moderate agreeableness and extraversion.

A multivariate analysis of variance was conducted, with personality type as the independent variable and COVID-19 information FOMO, resilience, anxiety, and depression as dependent variables (Table 4). The results showed that different personality types had significant differences in the levels of these four dependent variables (*p*s < 0.001).

### 3.4. Moderating Effects of Personality Types

Through model comparisons and multi-group analysis, moderated effects of personality types were found. We added the variable personality types to Model 1 and Model 2 and examined the effect of personality types in these two models.

#### 3.4.1. The Effects of Personality Types on the Relationships among COVID-19 Information FOMO, Resilience, and Depression

The results of the model comparison with depression as the outcome variable are listed below. First, based on Model 1, we added a categorical variable (personality types) to establish a configural invariance model and path equivalence model. The configural invariance model allowed all paths to be freely estimated for each personality type, while the path equivalence model meant that the structure paths of the three personality types were set equally. The results showed that the mediation model had differences across the three personality types (Δχ^2^ = 94.43, Δ*df* = 6, *p* < 0.001, ΔAIC = 82.43, ΔSSABIC = 69.85). Second, via further model comparisons, we found that the specific paths of resilience to depression and COVID-19 information FOMO to depression were moderated by personality types. Furthermore, a multi-group analysis showed that, among the three personality types, the effect of an undercontrolled personality was higher than the effect of an overcontrolled personality on the paths of resilience to depression and COVID-19 information FOMO to depression, and the adaptive effect was the lowest.

**Figure 4:**
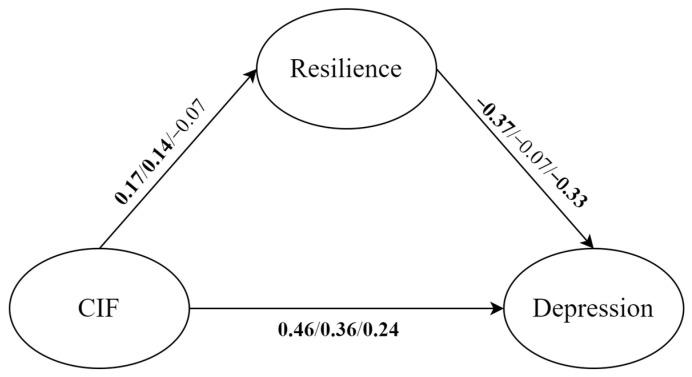
With depression as the outcome variable, mediation effect analysis results for undercontrolled, overcontrolled, and adaptive personalities, respectively. The path coefficients in bold mean *p* < 0.05. CIF: COVID-19 information FOMO.

Mediation effect analysis results for each personality type are presented as follows (Figure 4). For the undercontrolled personality, COVID-19 information FOMO positively predicted resilience (β = 0.17, *p* < 0.01, 95%*CI* = [0.07, 0.28]) and depression (β = 0.46, *p* < 0.001, 95%*CI* = [0.36, 0.55]), while resilience had a significant negative association with depression (β = −0.37, *p* < 0.001, 95%*CI* = [−0.47, −0.25]). For the overcontrolled personality, COVID-19 information FOMO positively predicted resilience (β = 0.14, *p* < 0.05, 95%*CI* = [0.03, 0.27]) and depression (β = 0.36, *p* < 0.001, 95%*CI* = [0.25, 0.45]), while resilience had no significant association with depression (β = −0.07, *p* > 0.05, 95%*CI* = [−0.19, 0.04]). For the adaptive personality, COVID-19 information FOMO positively predicted depression (β = 0.24, *p* < 0.01, 95%*CI* = [0.09, 0.36]), and resilience had a significant negative association with depression (β = −0.33, *p* < 0.001, 95%*CI* = [−0.49, −0.15]), while COVID-19 information FOMO had no significant link with resilience (β = −0.07, *p* > 0.05, 95%*CI* = [−0.20, 0.09]).

Under the influence of three personality types, the direct and indirect effects of all mediation paths were not the same as in Model 1. Overall, only undercontrollors’ indirect effects were significant (β = −0.06, *p* < 0.01, 95%*CI* = [−0.12, −0.02]), and the |ab/c’| was 14.0%. In addition, the undercontrolled personality had the greatest value of total effect (β = 0.40, *p* < 0.001, 95%*CI* = [0.30, 0.48]) among the three personality types, followed by the overcontrolled personality (β = 0.35, *p* < 0.001, 95%*CI* = [0.25, 0.44]) and adaptive personality (β = 0.26, *p* < 0.001, 95%*CI* = [0.10, 0.38]).

#### 3.4.2. The Effect of Personality Types on the Relationships among COVID-19 Information FOMO, Resilience, and Anxiety

The results of model comparison with anxiety as the outcome variable were as follows. The process of model comparison was similar to the above. First, we established the free estimation model and path equivalence model, compared the two model fit indices, and found that moderated effects of personality types existed in the mediated model (Δχ^2^ = 87.10, Δ*df* = 6, *p* < 0.001, ΔAIC = 75.10, ΔSSABIC = 62.51). Second, further model comparison and multigroup analysis indicated that, among the three personality types, the effect of undercontrolled personalities was higher than the effect of overcontrolled personalities in the path of COVID-19 information FOMO to anxiety, and the adaptive effect was lowest.

**Figure 5:**
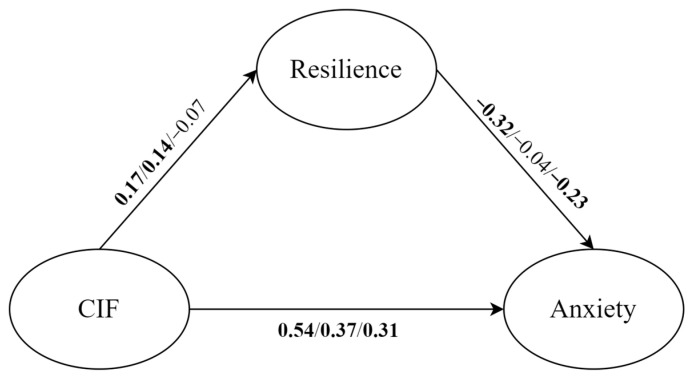
With anxiety as the outcome variable, mediation effect analysis results for undercontrolled, overcontrolled and adaptive personalities, respectively. The path coefficients in bold meant *p* < 0.05. CIF: COVID-19 information FOMO.

Mediated effect analysis results for each personality type are shown below (Figure 5). For undercontrolled personalities, COVID-19 information FOMO positively predicted resilience (β = 0.17, *p* < 0.01, 95%*CI* = [0.07, 0.28]) and anxiety (β = 0.54, *p* < 0.001, 95%*CI* = [0.44, 0.62]), while resilience had a significant negative association with anxiety (β = −0.32, *p* < 0.001, 95%*CI* = [−0.42, −0.20]). For overcontrolled personalities, COVID-19 information FOMO positively predicted resilience (β = 0.14, *p* < 0.05, 95%*CI* = [0.03, 0.27]) and anxiety (β = 0.37, *p* < 0.001, 95%*CI* = [0.27, 0.47]), while resilience had no significant association with anxiety (β = −0.04, *p* > 0.05, 95%*CI* = [−0.16, 0.08]). For adaptive personalities, COVID-19 information FOMO positively predicted anxiety (β = 0.31, *p* < 0.001, 95%*CI* = [0.17, 0.44]), and resilience had a significant negative association with anxiety (β = −0.23, *p* < 0.01, 95%*CI* = [−0.40, −0.07]), while COVID-19 information FOMO had no significant relationship with resilience (β = −0.07, *p* > 0.05, 95%*CI* = [−0.20, 0.09]).

Focusing on the role of the three personality types, the direct and indirect effects of all mediation paths were different from Model 2. Overall, only undercontrollors’ indirect effects were significant (β = −0.06, *p* < 0.05, 95%*CI* = [−0.11, −0.02]), and the |ab/c’| was 10.3%. Additionally, the undercontrolled personality also had the greatest value of total effect (β = 0.48, *p* < 0.001, 95%*CI* = [0.39, 0.56]) among the three personality types, followed by the overcontrolled personality (β = 0.37, *p* < 0.001, 95%*CI* = [0.26, 0.46]) and adaptive personality (β = 0.33, *p* < 0.001, 95%*CI* = [0.18, 0.45]).

## 4. Discussion

Due to the insufficient study on the effect of COVID-19 information FOMO on mental health (i.e., depression and anxiety) and the unclear mechanism underlying these relationships, the present study examined how COVID-19 information FOMO relates to depression and anxiety through resilience, as well as exploring the moderating role of personality types. We obtained three main findings. First, COVID-19 information FOMO is positively related to depression and anxiety. Second, resilience mediated the relationships between COVID-19 information FOMO and depression and anxiety. Third, personality type moderated the mediation models. In detail, the mediating role of resilience could only be found in the participants classified in the undercontrolled group, not the participants classified in the other two groups.

### 4.1. The Relationships between COVID-19 Information FOMO and Depression and Anxiety

This study revealed that COVID-19 information FOMO was positively related to depression and anxiety; thus, H1 was supported. That is, individuals with high levels of COVID-19 information FOMO tended to report high levels of depression and anxiety. This finding provides direct evidence about the relationship between COVID-19 information FOMO and mental health, which is in accordance with previous indirect evidence that indicates positive relationships between social FOMO and depression and anxiety [18,19]. The two probable explanations for these results are presented below. On the one hand, individuals with high COVID-19 information FOMO tend to report a high level of daytime tiredness; daytime tiredness is linked to mental health during COVID-19 [5,64]. Hence, individuals with high COVID-19 information FOMO experience high daytime tiredness, and daytime tiredness may cause mental health problems (e.g., depression and anxiety). On the other hand, individuals with more self-regulation tend to report better mental health and well-being during the pandemic [65]. Thus, high COVID-19 information FOMO may exceed the limits of self-regulation, leading to mental health problems increasing.

### 4.2. The Mediating Role of Resilience

One important finding of this study was that resilience mediated the relationships between COVID-19 information FOMO and depression and anxiety, which supports H2. This finding is similar to those of previous studies, in which resilience mediates the associations between negative conditions and mental health [20,25]. However, we found that the direct effects and indirect effects of COVID-19 information FOMO on depression and anxiety were opposite. The indirect effects reflected the suppressing effects [62]. After the variable of resilience was added to the mediation models, the direct effects of COVID-19 information FOMO on depression and anxiety were stronger than the total effects. This result of the present study partly differs from previous studies. Previous studies showed that the relationship between negative conditions (e.g., stressful COVID-19-related life events, pandemic fatigue) and resilience was negative [25,26], while we found that COVID-19 information FOMO was positively correlated with resilience.

The challenge model of resilience [66,67] provides a possible explanation for these results. This model explains that exposure to modest risks can enhance people’s abilities (e.g., resilience) and help them to overcome subsequent risks; for example, COVID-19-related stress may provide opportunities for post-traumatic growth [23]. In our study, we found a modest level of COVID-19 information FOMO among the participants (*M* ± *SD* = 2.94 ± 1.13). People with modest COVID-19 information FOMO like to search for information about the COVID-19 pandemic before exposure to a very high-risk environment. Hence, according to the challenge model of resilience, exposure to a risky environment during the COVID-19 pandemic may enhance individuals’ resilience in overcoming the risks. Therefore, the present study argues that COVID-19 information FOMO may improve individuals’ resilience, therefore reducing the risk of depression and anxiety. This result posits a double-edged-sword effect of COVID-19 information FOMO on mental health.

### 4.3. The Moderating Role of Personality Types

We found that Chinese undergraduates were classified into three personality types, of undercontrolled, adaptive, and overcontrolled, which supports H3. This classification is in line with previous studies [33,36,38]. Overall, this study enriches the research on personality psychology by adopting a person-centered approach.

Our findings showed personality types served as a moderator in the mediation models of COVID-19 information FOMO and depression and anxiety through resilience, which partly supports H4. Specifically, in terms of the direct effects, for undercontrollers, the relationships between COVID-19 information FOMO and depression and anxiety were strongest compared with the overcontrolled and adaptive groups. A possible explanation for this is that undercontrollers are at risk of the co-occurrence of internalizing and externalizing problems, while the adaptive group tends to have more prosocial behavior and less depression and anxiety [8,9,36,42]. Additionally, in terms of the indirect effects, only undercontrollers reported the indirect effects, while adaptive and overcontrolled groups reported no significant indirect effects.

The path coefficients from COVID-19 information FOMO to resilience were only significant in undercontrollers and overcontrollers. A possible reason for this is that the adaptive group tends to report the highest levels of resilience among individuals with the three personality types. Thus, for undercontrolled and overcontrolled groups, modest COVID-19 information FOMO gives them a chance to increase their levels of resilience, a possible way of promoting personality maturation.

The path coefficients from resilience to depression and anxiety were only significant in the undercontrolled and adaptive groups. In our study, we found that neuroticism and extraversion had stronger correlations (|r| > 0.3) with depression and anxiety than the other three personality traits (|r| < 0.3), so this explanation is mainly presented from the perspective of neuroticism and extraversion. Moreover, previous research showed that neuroticism might attenuate the negative effect of resilience on depression [68] and anxiety [69], while extraversion might enhance the negative effect of resilience on depression [68]. Compared to overcontrollers, undercontrolled and adaptive groups had lower neuroticism and higher extraversion, so they tended to report stronger relationships between resilience and depression and anxiety. Nonetheless, for overcontrollers, the positive effects of neuroticism may counteract the negative effects of resilience on depression and anxiety. When we take no account of the slightly negative effect of low extraversion on depression and anxiety, the links between resilience and depression and anxiety are not significant. Therefore, personality types moderated the mediation models showing that the indirect effects of resilience were only significant in the undercontrolled group rather than in the other two groups.

### 4.4. Implications

The current study has both important theoretical and practical implications. For the theoretical implications, our work is the first study examining the relationships between COVID-19 information FOMO and depression and anxiety, which fills the gap left by previous studies on the relationship between information FOMO and mental health, especially during a major public health emergency (e.g., the COVID-19 pandemic). It emphasizes the need for greater attention to the mental health of undergraduates, focusing on COVID-19-related information. Also, the suppressing effects provide evidence for the establishment of mental-health-protective mechanisms of resilience in the COVID-19 pandemic and enrich the connotations of challenge model of resilience. Meanwhile, the moderation effect analysis provides a boundary condition for the mediation models. That is, the mediating effects of resilience are only found in overcontrollers, instead of in the undercontrolled and adaptive groups.

Moreover, the present study has practical implications. This study provides us with guidance to make personalized suggestions to intervene and alleviate the negative outcomes resulting from COVID-19 information FOMO. First, colleges can provide customized group psychological counseling programs for overcontrollers to improve their resilience. Second, the state and government should compile a reporting strategy for possible mass trauma incidents like COVID-19 and collaborate with all facets of the community to efficiently disseminate relevant scientific information. Third, the state and government should also be concerned about the mental health of the population and offer psychological aid, such as setting up several psychological hotlines, in the event of a large public health disaster (e.g., the COVID-19 pandemic). By taking these steps, college students’ sense of security can be increased, and information FOMO and the mental health issues it might create can be decreased.

### 4.5. Limitations and Future Studies

The current study findings should be considered in light of three limitations. First, this study was a cross-sectional study using the questionnaire method. In our study, we did not measure participants’ COVID-19 information FOMO at the early stage of the pandemic; therefore, changes in the potential effect of COVID-19 information FOMO from the early pandemic to the data collection date for our results cannot be controlled for. This limitation may reduce our results’ validity. To address this limitation, we encourage other scholars to adopt experimental or longitudinal research methods in the future. Second, the scales used in the present study were all self-reported, potentially giving rise to response bias. Therefore, multiple sources and methods should be used to collect data in future research. Third, Cronbach’s alpha for extraversion is 0.54, which was not acceptable. This limited the conclusions regarding the relation to extraversion. Fourth, COVID-19 information FOMO included only three general questions and did not break down COVID-19 information FOMO into FOMO categories, such as medical information, impact news, social impact, etc. The guidance that extended to public health messaging was weakened. Fifth, the participants in the present study were Chinese undergraduates, which may shrink the external validity. Thus, generalizations of the results should be made carefully.

## 5. Conclusions

The present study examined the relationships between COVID-19 information FOMO and depression and anxiety, as well as the mediating role of resilience and the differences in personality types. In summary, this study suggests that COVID-19 information FOMO was positively related to depression and anxiety through resilience. Additionally, personality types moderated the mediation models, in which the mediating effect was significant among participants who were classified into the undercontrolled group. We highlight that, in responding to a major public health emergency (e.g., the COVID-19 pandemic), attention should be paid to public mental health.

## Figures and Tables

**Table 1:** Descriptive statistics and correlation matrix of all variables.

Variable	1	2	3	4	5	6	7	8	9
1. CIF	1								
2. Resilience	0.15 ***	1							
3. Neuroticism	0.35 ***	−0.08 **	1						
4. Conscientiousness	0.15 ***	0.63 ***	0.00	1					
5. Agreeableness	0.06 *	0.55 ***	0.02	0.52 ***	1				
6. Openness	0.30 ***	0.57 ***	0.14 ***	0.53 ***	0.40 ***	1			
7. Extraversion	−0.13 ***	0.23 ***	−0.48 ***	0.17 ***	0.12 ***	0.19 ***	1		
8. Depression	0.38 ***	−0.21 ***	0.55 ***	−0.15 ***	−0.14 ***	0.04	−0.38 ***	1	
9. Anxiety	0.43 ***	−0.14 ***	0.54 ***	−0.08 **	−0.07 **	0.11 ***	−0.34 ***	0.89 ***	1
*M*	2.94	3.08	3.75	4.54	4.81	4.17	3.43	1.92	1.99
*SD*	1.13	0.44	1.13	0.80	0.75	1.04	0.93	0.73	0.72

Note. CIF = COVID-19 information FOMO. * *p* < 0.05; ** *p* < 0.01; *** *p* < 0.001.

**Table 2:** Standardization of direct effects and indirect effects in the model.

Outcome		Effect	*SE*	95%*CI*	
Depression	Indirect effect	−0.05 ***	0.01	−0.04	−0.02
	Direct effect	0.45 ***	0.03	0.22	0.30
	Total effect	0.40 ***	0.03	0.20	0.27
Anxiety	Indirect effect	−0.04 ***	0.01	−0.03	−0.01
	Direct effect	0.49 ***	0.03	0.23	0.30
	Total effect	0.46 ***	0.03	0.21	0.28

Note. Bootstrap sample size = 5000. CIF: COVID-19 information FOMO. *** *p* < 0.001.

**Table 3:** Criteria for latent profile models of personality types.

	AIC	BIC	SSABIC	Entropy	LMR-LRT (*p*-Value)	BLRT (*p*-Value)
2-Class	19,677.82	19,762.20	19,711.38	0.68	<0.001	<0.001
3-Class	19,019.36	19,135.39	19,065.50	0.77	<0.001	<0.001
4-Class	18,795.30	18,942.97	18,854.02	0.82	0.29	<0.001

**Table 4:** Means and standard deviations of each personality type.

	CIF	Resilience	Depression	Anxiety
Overcontrolled	3.50 ^a^ (1.10)	3.26 ^b^ (0.34)	2.22 ^a^ (0.85)	2.32 ^a^ (0.85)
Undercontrolled	2.71 ^b^ (0.99)	2.78 ^c^ (0.37)	2.00 ^b^ (0.59)	2.02 ^b^ (0.58)
Adaptive	2.57 ^b^ (1.14)	3.38 ^a^ (0.30)	1.29 ^c^ (0.29)	1.42 ^c^ (0.33)
*F*	101.73 ***	429.35 ***	207.83 ***	190.96 ***
partial η^2^	0.12	0.37	0.22	0.21

Note. CIF: COVID-19 information FOMO. Different letters represent significant differences (*p* < 0.05). *** *p* < 0.001.

## Data Availability

The data that support the findings of this study are available on request from the corresponding author.
